# Raw diffraction data are our ground truth from which all subsequent workflows develop

**DOI:** 10.1107/S2059798322003795

**Published:** 2022-05-18

**Authors:** John R. Helliwell

**Affiliations:** a Department of Chemistry, University of Manchester, Manchester M13 9PL, United Kingdom

**Keywords:** raw diffraction data, workflows, FACT and FAIR, versions of record

## Abstract

Defining best practice in science is challenging. International consensus is facilitated by the International Science Council via its members such as the International Union of Crystallography (IUCr). The crystallographic community has many decades of tradition linking articles with the underpinning data, and is admired across all sciences accordingly. Crystallography has always been at the forefront of harnessing new technology in the service of consensus. Technology has provided new vast data-archiving opportunities, allowing the preservation of raw diffraction data, along with article and database depositions of a model’s coordinates and associated structure factors. The raw diffraction data, which can now be preserved, are the ground truth from which all subsequent workflows develop. Journal editorial boards provide a practical forum for setting the criteria to decide if a study’s files are truly the version of record. Within that, reality involves a variance of reasonable workflows. But what is a reasonable variance? Workflows must be detailed carefully by authors in explaining what they have done. There is a great, and increasing, diversity of macromolecular crystallography analyses, and yet an increased constraint on how much can be written in an article about the workflow used. Raw data provide the ultimate reproducibility evidence. A part of reproducibility and replicability is using an agreed vocabulary; the meaning of words such as precision and accuracy and, more recently, the confidence of a protein structure prediction should feature in approaching ‘truth’.

## Abstract 2

Defining best practice in science is challenging. International consensus is facilitated by the International Science Council via its members such as the International Union of Crystallography. The IUCr Journals editorial boards are a practical forum for setting the criteria to decide if a study’s files are truly the ‘version of record’. Within that, reality involves a variance of reasonable workflows. Workflows must be detailed carefully by authors in explaining what they have done.

## 1. Introduction: the nature of the challenge to reproducibility and replicability in our science

To define the topic of what is best practice in one field of science such as crystallography first raises more general questions on what is best practice in science. Namely, is objectivity, and truth, possible? A publication is an important narrative of the work done and interpretations made by researchers securing a scientific discovery. As The Royal Society neatly states though, *nullius in verba* (take nobody’s word for it), whereby the role of the underpinning data is paramount. Therefore, the objectivity that preserving the data within an article provides is due to readers being able to check the calculation decisions of the authors. The wider science data scene introduced the FAIR data accord, namely, that data are Findable, Accessible, Interoperable and Reusable (Wilkin­son *et al.*, 2016 ▸). Some social scientists also emphasize more than FAIR being needed, the data should be FACT, which is an acronym meaning Fair, Accurate, Confidential and Transparent (van der Aalst *et al.*, 2017 ▸), this being the issue of ensuring reproducibility not just reusability. Confidentiality of data is not likely to be generally relevant to our data. The important exception is during pre-publication peer review when authors rightly expect their narrative and their underpinning data to be treated confidentially during a journal’s peer-review procedures. I have discussed where crystallography sits in this wider science landscape. Indeed, it sits well within many of its traditions, especially the exemplary chemical crystallography peer-review procedures for journals such as *Acta Crystallographica Section C* (Helliwell, 2019 ▸).

The role of raw data has been identified in the field of machine learning as their ‘ground truth’; this is also a useful concept for science conducted by a person. New vast data-archiving opportunities allow us to preserve our raw diffraction data, along with our article and database depositions of a model’s coordinates and associated structure factors. The raw diffraction data are our ground truth from which all subsequent workflows develop.

The practicalities of having effective raw data reuse rely, as with processed diffraction data and the derived molecular model, on the metadata being at least adequate. In addition, it needs to be feasible to move individual raw diffraction data sets around over the network. Both these points, rich metadata and network data transfer feasibility, have been considered in detail at the IUCr Diffraction Data Deposition Working Group (DDDWG) Workshops and several associated publications. These are summarized in the DDDWG Final Report at https://www.iucr.org/resources/data/dddwg/final-report; good examples are Tanley *et al.* (2013 ▸), Terwilliger & Bricogne (2014 ▸), Kroon-Batenburg & Helliwell (2014 ▸) and Kroon-Batenburg *et al.* (2017 ▸). The instrument metadata core list is described in the IUCr DDDWG Final Report and subsequent discussion has identified the diffraction image beam centre as being prone to error. Such an error can derail reflection indexing of course. Beamline staff need to be vigilant to notice beam-centre errors in metadata. The metadata for a sample is another category of challenge. For raw data, PDB depositions and a publication on a particular study, the sample under discussion will be clear. The challenge here is not with published studies and their data but with the release to the public of unpublished raw data. This is starting in earnest as synchrotron facilities such as Diamond, ESRF and Soleil have established data policies which envisage release three years after measurement (Helliwell, 2022 ▸). Effective data reuse of unpublished raw data depends on adequate metadata about the sample being made available. Ideally this should include a release of the beamtime proposal document at the same time as the release of the raw data; an abstract alone may well not be adequate for effective data reuse.

Let us also reflect on crystallographers’ efforts over many decades. We can start by citing W. L. Bragg’s paper on the first ever crystal structure of sodium chloride (Bragg, 1913 ▸). He achieved this by comparing the Laue diffraction patterns of several different alkali halide crystals especially comparing those from NaCl and KCl. The latter involved nearly isoelectronic elements, in fact identical as it turned out, the crystal structure involved isoelectronic K^+^ and Cl^−^ ions. Bragg deduced the two interpenetrating face-centred cubic lattices layout of Na^+^ and Cl^−^ ions in NaCl, and which reduced to a simple primitive lattice layout of ions in KCl. Bragg included 15 Laue diffraction patterns in his article. In his 1975 book looking back at his career, The Development of X-ray Analysis (Bragg, 1975 ▸), he made it clear that he could not be sure of his NaCl crystal structure without his use of his father’s (W. H. Bragg) monochromatic spectrometer (we refer to this now as a diffractometer). His 1913 paper explicitly showed the intensity reflection rocking curve of these data. W. L. Bragg gave us then a clear view of the importance of the diffraction data being part of our articles, or as we would do now by linking to it. Bragg also gave us the simple maxim that crystal structure analysis gives us a map of the structure (Bragg, 1968 ▸). In effect he was telling us that we can see atoms.

**Figure 1:**
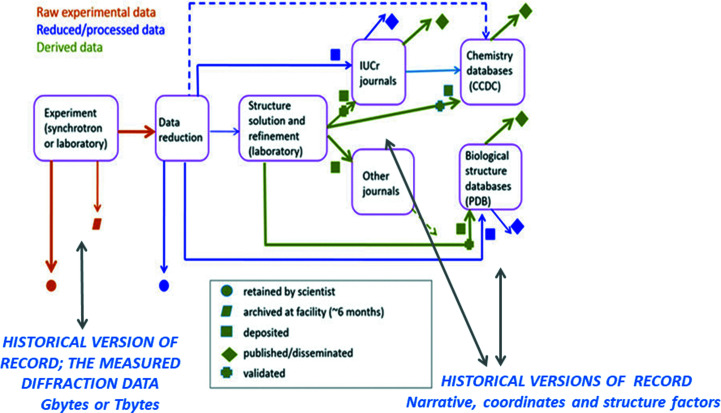
Crystallography workflows [developed from Hall & McMahon (2016 ▸) and Kroon-Batenburg *et al.* (2017 ▸) with thanks to Brian McMahon]. There are two specific moments of ‘historical record’; the measured calibrated raw data and the researchers’ finalized narrative along with their ‘best’ molecular structure model and processed structure factors.

Today, looking at the whole vista of what a crystal’s internal layout is in terms of order versus disorder (static and dynamic) and/or the challenges of making our measurements, the life of the crystallographer is not so simple. Many tools have been developed both in terms of instrumentation, harnessing the different probes (X-rays, neutrons, electrons and NMR) and computational software. Thus, to a good degree, community best practices have emerged for the typical situations and with them the most likely workflows. Perhaps unsurprisingly, the workflows of chemical crystallography have had a head start by several decades over fields such as biological crystallography. Materials crystallography, meaning functioning materials, are somewhere in between chemistry and biology as practiced disciplines as they aim at directly probing specific functions and so further diffraction tools are needed for studies of samples in their functioning states. These sample states (liquid, amorphous or powder) have led to techniques to study them cojoined with crystallography such as small-angle scattering, pair distribution functions or powder diffraction profiling. Biological crystallography seems to have been especially challenging as its overall aim is to learn *what is the structural chemistry of the living organism at its temperature and pressure* (this is the article title I used in Helliwell, 2020 ▸). This latter sentence seems to be a statement of the obvious as that is indeed what Max Perutz did in his Nobel pioneering protein crystallography studies of haemoglobin in its functioning states of loading or unloading oxygen; ‘haemoglobin the molecular lung’ as he later put it (Perutz, 1971 ▸). Today, in 2022, our macromolecular crystallography workflows seem rather more complicated. These workflows are, in principle, coherent (Fig. 1 ▸) and yet, of course, there are rather diverse software options, as can be simply checked at any given time using the software-use statistics at the PDB (https://www.rcsb.org/stats/distribution-software). There has also been a diversity of instrumentation hardware over the past 50 years. Of course, there is the diversity of the diffraction probes of the structure of matter itself; electrons, neutrons and X-rays. Within X-rays alone the detector hardware has evolved substantially: film, TV, multiwire proportional chamber, image plate/storage phosphor, CCDs (and with differing counting chains) and pixel detectors. This evolution has been guided by the measurement physics properties of parameters such as detector quantum efficiency for each device compared with the ideal of a perfect detector *i.e.* noise free across all the intrinsic intensity range of weak, medium and strong Bragg reflections. This aim must be combined with a uniform area and linear response of the detector for all choices of X-ray wavelength such as with synchrotron radiation and for high local and global count rates (see Chapters 4 and 5 of Helliwell, 1992 ▸). The overarching aim is precise measurements from each type of device.

In judging whether an article about a study and the associated data files are to be admitted as versions of record, the IUCr’s chemical crystallography journal editors, and their referees, decided that they needed to see all these items together and make their own calculations if they were curious and wanted to check the data. They also realized, as a community, that there was the chance to perform a range of checks and these became embedded in the checkCIF procedure, now comprising over 400 checks on the consistency and integrity of crystal structure determinations, with a range of alerts A, B and C indicating the severity of any problems. A website offering a pre-article-submission checkCIF service is provided by the IUCr (https://checkcif.iucr.org). The editors of the IUCr biological crystallography journals have adopted a similar procedure. For several years now I have insisted on having access to the coordinates, structure factors and PDB validation report before I would referee an article; I wrote up my experiences in Helliwell (2018 ▸), offered as data science skills guidelines. A key part of this, I argued, is to recognize a variance of the calculations in apparently identical workflows, but arising from using different computer programs, because as a referee I might well use different programs to those used by the authors. The recent editorial of the IUCr Journals biological main editors describes the important change to its validation procedures for its submissions (Baker *et al.*, 2021 ▸).

Another source of variance is due to the lack of agreed consensus of different members of the community. Areas of variance could include items such as the criteria for when a bound water is observed or not. Splendid visualization tools exist in programs such as *Coot* (Emsley & Cowtan, 2004 ▸; Emsley *et al.*, 2010 ▸). But what criteria for their observability should be insisted on? It is to be expected that the IUCr Journals editorial boards for biological crystallography would arrive at an agreed ruling on such a matter where it was specifically important to a given article. It is the bound waters at a ligand binding site which are displaced, and form an entropic benefit, or are the glue through which a ligand binds itself to a protein receptor site. These bound waters as a category are therefore especially important. This is one example of the nature of the challenge to knowing the reproducibility in a study and the replicability of it in other studies in our science of crystallography. Peer review to determine versions of record has led to the wwPDB introducing the chance for depositors to reversion their deposited files. There is also, however, the case of PDB deposits without an associated publication but again reversioning will be a useful tool for such depositors.

There are also some bigger questions:

Is there a unique *best* practice in computational crystallography, or is a variance of interpretations *reality*?
What about making a *hypothesis after the research is known* (HARKing) or is such a situation just fortunate serendipity?

Suffice to say, our workflows must be detailed carefully in explaining what we have done, and our raw data are a study’s *ground truth*.

At the time of writing this article, the *de facto* best practice in biological crystallography is defined in the Notes for Authors for *Acta Cryst. D* (https://journals.iucr.org/d/services/notesforauthors.html).

## 2. Historically how have we described our workflows, and what do we do today?

**Figure 2:**
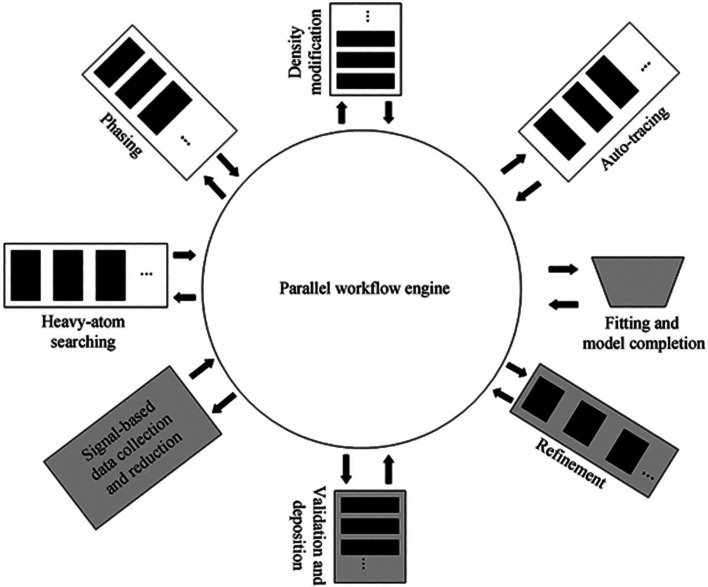
An example of an overall, MX structure determination workflow from the mid-2000s. From **SGXPro*: a parallel workflow engine enabling optimization of program performance and automation of structure determination* by Fu *et al.* (2005 ▸).

We used to publish workflow details in the main text not just in a supplementary document. These were assembled manually. By the mid-2000s a strong interest of the community, perhaps driven by the concept of the structural genomics projects to primarily expand the protein fold space, was to automate workflows. Within this, the parallelization of the different stages of macromolecular crystallography (MX) arose. An example is shown in Fig. 2 ▸; I note that in this figure there is no explicit mention of a journal, and its peer-review procedure, in the pipeline. Clearly, I think this is a step that must be part of our striving for best practice (Helliwell, 2018 ▸). I stress at this point that the PDB does a very good job with its validation report guiding authors and referees, like the IUCr checkCIF report, in the standardized checks of and queries on a crystal structure. But it makes no judgement, for example, of the selection of bound waters by the authors nor indeed offers any comment. Recently the PDB has extended its validation report to assess the fitting of ligands by offering pictures of the difference map peaks on a ligand and that might thereby lead to an improved model for the ligand. Other significant peaks in the difference map are not commented on; *Coot* already provides a difference map peaks list which is very helpful to referees and the editor. The *Acta Cryst. D* Notes for Authors ask for such information to be included in the article.

So, today, in terms of reporting a structure-based research study, the precision of the crystal structure can be definitive if there is a full description of the workflow in the article and the assessment by the journal of the underpinning data files, assisted by the PDB’s validation report. This journal peer review can now be extended to the raw diffraction data as not only are data archives of sufficient capacity available but also network transfer speeds allow ready transfer of those ground truth raw data to the journal and onwards to the referees.

## 3. Challenges to recording workflows

With ‘nightly software builds’ on GitHub it is challenging for an author to provide a journal editor and their referees with the exact version of the various software/programs that they used in their workflow, often much earlier (even years before) than their article preparation.

Also, some programs can be so detailed in their sophistication I, at least, cannot always reproduce my own workflow *e.g.* when I come back to them later. Of course, these are only in the especially challenging research projects. More generally, and to focus on a quite topical area of the CCP4 bulletin board (CCP4bb) in the last year, I elaborate in the next section on difference maps.

### 3.1. Choosing a difference map such as for investigating a ligand

There are various options for a structural crystallography researcher in this specific domain. This was the focus of a quite intense CCP4bb debate in late 2020/early 2021 (*e.g.* involving Dale Tronrud, Rob Nicholls and myself) about *Coot*’s sharpening/blurring map tool, and in particular the debate raised the question ‘is *hypothesis after the research is known* (HARKing) a problem in MX?’.

The fulcrum of this debate was balancing intuition and false preconception. As Nicholls (2017 ▸), building on Nicholls *et al.* (2012 ▸), states on behalf of intuition:

Another feature that can help one to gain intuition is map blurring (found under ‘Map Sharpening’ in the ‘Calculate’ menu in *Coot*), which involves adding a very high *B* factor to the density map in order to give higher weight to the lower resolution reflections. This can provide evidence for the presence of structure in the crystal that is currently missing from the model, especially in cases where the ligand is particularly flexible. Indeed, viewing different types of density maps can facilitate the extraction of as much information as possible.

But there are indeed quite a range of maps available to the researcher today, and not all are shown in a paper but instead maybe one is chosen that perhaps most favourably resembles the researcher’s preconceptions; a tendency emphasized by Rupp (2010 ▸).

Types of difference map include:

(1) A ‘before adding the ligand at all’ type of omit map, after refinement of the protein alone.
(2) An omit map calculated after having included and refined the ligand in the model:(i) A ‘feature enhanced map’ (described in Afonine *et al.*, 2012 ▸).(ii) A ‘polder map’ (described in Liebschner *et al.*, 2017 ▸).(iii) A *CCP*4 omit map (Winn *et al.*, 2011 ▸).(iv) A *Phenix* omit map (Afonine *et al.*, 2012 ▸).(v) A composite omit map (Winn *et al.*, 2011 ▸, Afonine *et al.*, 2012 ▸). (Interestingly the two software packages take very different times to make this calculation.)

The above options for the choice of difference map are also interrelated with how we estimate the phases of our structure factors, initially experimental but then increasingly calculated phases, as our model improves. Within this context looms the worry of model bias. Indeed, the concept of an omit map for a ligand seems a flawed one to me by which I mean once included in the model can one ever remove the worry of that particular-model bias? Surely, the best difference map is before any ligand is included. A similar semantic question arises from the terminology ‘validation’ report by which I mean that surely a better approach would be a report on what is invalid. The chemical crystallography checkCIF report does do this by raising alerts A, B or C as to what might be invalid in a chemical crystal structure.

A particular challenge is the situation of a lower occupancy ligand. The clarity of the difference density is vulnerable to weak density in the situation where waters are displaced. Also, in the situation of cryocrystallography, Halle (2004 ▸) has identified this as being a category of possible structural artefact, which at a higher temperature such as room or physiological temperature could disappear. This specifically highlights the situation where we can ensure our cryocrystal structure is precise but actually is it a realistic, *i.e.* accurate, model *in vivo*?

Overall, of course even if we choose a room temperature for our diffraction measurements our macromolecule is one of a very large number packed into an ordered crystalline array. At this point, in striving for accuracy, we need a different method altogether (in the eyes of a trained physicist like myself) such as our macromolecules at least being free of lattice effects, as in a solution. However, an interesting middle ground in terms of methodology is where we adopt a direct time-resolved measurement in a functioning crystal. In my laboratory’s studies of the enzyme hydroxymethylbilane synthase (Helliwell *et al.*, 1998 ▸) we could see in our time-resolved difference electron-density snapshots a growing electron density adjacent to the essential cofactor and projecting out into solvent in the crystal (see also Nieh, 1997 ▸). Unfortunately, the details of this extended electron density were unclear which we presume were due to a range of structural dynamics of the growing pyrrole chain. I assert that our study is structurally accurate but not precise!

At the most basic level, when we deposit our crystal structure molecular model at the PDB, we must offer a *single molecule*. The crystal structure, though, could be very likely to include disordered atoms, in both statically diverse and dynamically changing positions. Moreover, there are likely to be displacement waves (phonons) in the crystal. Crystal perfections and imperfections, as well as macromolecular crystallization, are described in detail in the book by Chayen *et al.* (2010 ▸). Within our one model we can however readily determine the atomic displacement parameters (ADPs, also known as the atomic *B* factors) which are usually isotropic but if a sufficiently high diffraction resolution is achievable then these ADPs can be determined as anisotropic ellipsoids and even modelled by aspherical scattering factors in very favourable cases. The measurement of diffraction data sets at multiple temperatures allows a determination of the dynamic as distinct from the static disorders. Incomplete treatments, formally, are bad for precision, *i.e.* the best possible precision is not reached.

## 4. Are some workflows easier to record than others?

Large-scale facilities are perhaps accustomed to automation these days, and this includes workflows. One such example is that of Brockhauser *et al.* (2012 ▸) at the ESRF who offer ‘example workflows designed and implemented using DAWB are presented for enhanced multi-step crystal characterizations, experiments involving crystal re­orientation with kappa goniometers, crystal-burning experiments for empirically determining the radiation sensitivity of a crystal system and the application of mesh scans to find the best location of a crystal to obtain the highest diffraction quality’.

For which steps might it be hard to record workflows? The molecular graphics stage involves going back and forth between modelling and refinement multiple times. Therefore, recording this workflow might be more challenging than recording the data collection workflow (Rob Nicholls, personal communication).

## 5. A part of precision and accuracy, as well as reproducibility and replicability, is using an agreed vocabulary

The meaning of words such as precision and accuracy, and more recently the confidence of a protein structure prediction, should feature in approaching *truth*, the overall objective in our subjective efforts. Cruickshank (1999 ▸) introduced the diffraction precision index (the DPI) for macromolecular crystallography thereby putting the precision of a biological crystal structure on a clear basis. By extending this (Kumar *et al.*, 2015 ▸) to individual atoms it is possible to properly label the precision of a non-bonded interaction in published figures (Gurusaran *et al.*, 2014 ▸). An *accuracy indicator* for a biological structure can be combined with a functional assay or its predictive force. Of course, what was not modelled shown by the difference Fourier map is of keen interest, or should be, to a journal in its peer review evaluation. For journals, those putting in the extra effort to ‘scrutinize article with data’ (Baker *et al.*, 2021 ▸) should be assigned a gold star, and thereby be a much better accolade than the impact factor. The growing availability of the archived raw data means that the overall quality, *i.e.* precision, of the structural models in the PDB may be improved with raw-data reprocessing, but this does not imply necessarily a change in the functional interpretation. I have also proposed that the PDB metrics, the slider diagram, focused on the precision of a model, could have added to those the details of physiological relevance of a biological function assay. These would then span not only model precision, the current situation, but also then the accuracy of a study.

In terms of vocabulary one of the consistent misuses is in predicted structures being defined as accurate. There is not a clear level of precision at the atom-by-atom detailed level *i.e.* akin to a diffraction precision index (Cruickshank, 1999 ▸; Gurusaran *et al.*, 2014 ▸; Kumar *et al.*, 2015 ▸). However there is reason for hope: DeepMind’s blog https://deepmind.com/blog/article/AlphaFold-Using-AI-for-scientific-discovery-2020 states that they are ‘indebted to…not least the experimentalists whose structures enable this kind of rigorous assessment’. In the absence of the experimental structure what is assessment then? It cannot be accuracy or precision, but it can clearly, like any modelling, be a confidence index, which can be overall and locally in the case of a protein structure. Tom Terwilliger (personal communication) has usefully emphasized that the prediction is a *hypothesis*.

What is the difference between the words reproducibility and replicability? A recent USA National Academies’ Report (2019 ▸) explores these two words in detail, a report prompted by the ongoing debate about a ‘reproducibility crisis in science’. We seek firstly to *reproduce* for ourselves the authors’ analyses from the authors’ data, maybe with our own workflow or the authors’ workflow and thus establish a variance of the results and outcomes based on the original data. We then design our own experiments, measure our own data and see if we *replicate* the first study’s results and conclusions.

## 6. Conclusions

My first overall conclusion is that it is important to measure the right thing (such as at the appropriate pH if one is especially interested in protonation states). This requirement may not be possible, *e.g.* if crystallization occurs far away from the *in vivo* pH. Secondly, to move beyond precision and via accuracy to come as close to truth as possible, one must try to combine all the techniques one can. I offer a wide range of examples in Helliwell (2021 ▸). My third overall conclusion is that authors should ensure that there is a detailed description of the workflow used and provide the journal editor with access to all the files that document a study: as well as the article, the coordinates file, the structure factors file, in addition to the PDB validation report and, in the future, the raw diffraction data.

The above three overall conclusions may be viewed as idealistic because there are challenges. Workflows do vary and variances must then be allowed for, but can there be an agreement on what variances are allowable? I have highlighted the choosing of reliable bound waters and at *in vivo* relevant conditions, and assessing and documenting a difference map ‘uninterpreted peaks’ list.

Overall, authors and readers will also look for consistency in the application of a journal’s notes for authors across multiple articles and including a consistent vocabulary across different editors.

In terms of an expected increasing role of raw diffraction data in the FAIR era envisaged in the IUCr DDDWG Final Report, several practical measures are well under way.

(i) Led by the IUCr Commission on Biological Macromolecules, chaired by Wladek Minor, IUCr Journals now state ‘for articles describing a new structure or a new method tested on unpublished data, authors are recommended to make arrangements for their original raw diffraction data to be archived in a repository that assigns a digital object identifiier (DOI) to the data. The assigned doi should be provided during the submission process and a link from the article to the data will be made upon publication’.
(ii) *IUCrData* has launched a new section called Raw Data Letters led by the new Main Editor Loes Kroon-Batenburg and within which a checkCIF for raw data has been developed to achieve robustness of raw diffraction data metadata. The wider utilization of checkCIF for raw data by such as the synchrotron data archives is expected to help with the robustness of their metadata and thereby their raw diffraction data reuse.
